# Age groups and spread of influenza: implications for vaccination strategy

**DOI:** 10.1186/1471-2334-10-106

**Published:** 2010-04-30

**Authors:** Ying-Hen Hsieh

**Affiliations:** 1 Department of Public Health and Center for Infectious Disease Epidemiology, China Medical University, Taichung, Taiwan

## Abstract

**Background:**

The unpredictable nature of the potentially devastating impact of 2009 pH1N1 influenza pandemic highlights the need for pandemic preparedness planning, where modeling studies could be most useful for simulations of possible future scenarios.

**Methods:**

A compartmental model with pre-symptomatic and asymptomatic influenza infections is proposed which incorporates age groups as well as intervention measures such as age-specific vaccination, in order to study spread of influenza in a community.

**Results:**

We derive the basic reproduction number and other effective reproduction numbers under various intervention measures. For illustration, we make use of the Pneumonia and Influenza (P&I) mortality data and vaccination data of the very young (age 0-2) and the very old (age >64) during 2004-2005 Taiwan winter influenza season to fit our model and to compute the relevant reproduction numbers. The reproduction number for this winter flu season is estimated to be slightly above one (~1.0001).

**Conclusions:**

Comparatively large errors in fitting the P&I mortality data of the elderly (>64) were observed shortly after winter school closings in January, which may indicate the impact of younger, more active age groups transmitting influenza to other age groups outside of the school settings; in particular, to the elderly in the households. Pre-symptomatic infections seemed to have little effect on the model fit, while asymptomatic infection by asymptomatic infectives has a more pronounced impact on the model fit for the elderly mortality, perhaps indicating a larger role in disease transmission by asymptomatic infection. Simulations indicate that the impact of vaccination on the disease incidence might not be fully revealed in the change (or the lack thereof) in the effective reproduction number with interventions, but could still be substantial. The estimated per contact transmission probability for susceptible elderly is significantly higher than that of any other age group, perhaps highlighting the vulnerability of the elderly due to close contacts with their caretakers from other age groups. The relative impact of targeting the very young and the very old for vaccination was weakened by their relative inactivity, thus giving evidence of the lack of impact of vaccinating these two groups on the overall transmissibility of the disease in the community. This further underscores the need for morbidity-based strategy to prevent elderly mortality.

## Background

In the spring of 2009, the novel H1N1 influenza virus first emerged in Mexico and later spread widely throughout the world within just a few months. The World Health Organization (WHO) announced on June 11 the start of 2009 influenza pandemic [1], and further issued an advisory on August 28 for countries in the northern hemisphere to prepare for a second wave of pandemic spread in the coming fall/winter [2]. As of November 8, more than 206 countries and overseas territories or communities worldwide have reported laboratory confirmed cases of the pandemic pH1N1 virus, including over 6250 deaths [3].

To lessen the severity of this pandemic, developing an effective flu vaccine and a global vaccination strategy is considered to be among the most important medical interventions [4]. However, to have the greatest impact, pandemic vaccines need to be available quickly and in large quantities, and to be delivered to the population optimally. Moreover, vaccines against a novel pandemic strain might take up to six months to manufacture and deliver, even in developed countries [5]. Given the potential threat of drug-resistance resulting from widespread use of antiviral treatment against pandemic flu, vaccine appears to be our primary weapon to prevent and to mitigate a pandemic. However, in addition to the need to consider the logistics of implementing large-scale vaccination, distinctly different age-specific mortalities had also been observed during some past flu pandemics (e.g., in 1918 [6]), which require different priorities when large-scale vaccination is to be implemented.

Moreover, vaccine for influenza is known to have different efficacy (i.e., reduction in the number of infectives) and effectiveness (i.e., reduction in symptomatic case number) for different age groups, see e.g., [7-9]. Setting priority for vaccination by targeting age groups most vulnerable (the elderly, infants, etc.) to prevent mortalities is commonly employed in most countries. However, when vaccinating those at greatest risk of mortality becomes impractical (if, e.g., medical care is relatively inaccessible) or inefficient (if, e.g., immune response is deficient), targeting those most likely to expose them to infection might be more preferable [10]. In this way, the very young and the old might be better protected by vaccinating those who are most likely to be in contact with them (thereby reducing their risk of exposure), rather than by being vaccinated. Comparison of influenza mortality among elderly Japanese during time periods when schoolchildren were and were not vaccinated suggests that the infected children pose a risk to others [11], including the elderly. Moreover, several past US experiences (as summarized in [12]) also are consistent with this conclusion. Nonetheless, influenza policymakers have typically advocated protecting those individuals of ages 6-24 months and >65 years directly.

Bansal et al. [13] recently carried out a comparative analysis of two classes of suggested vaccination strategies, namely, the mortality-based strategies that target the high-risk populations and the morbidity-based strategies that target the high-prevalence populations, by applying the methods of contact network epidemiology to a model of disease transmission in a large urban population. Using a range of mortality rates reported previously for past influenza epidemics and pandemics, they concluded that the optimal strategy depends critically on the viral transmission level (or reproduction number) of the virus. That is, the morbidity-based strategies outperform the mortality-based strategies for moderately transmissible strains, while the reverse is true for highly transmissible strains. However, they also cautioned that when information pertaining to viral transmission rate of a particular disease and the frequency of new introductions into the community prior to an outbreak is unreliable or not available, a mortality-based vaccination priority is recommended. This further demonstrates the importance of targeting and, moreover, the uncertainty surrounding this issue.

To further the uncertainties regarding influenza pandemic preparedness planning, it is widely believed that asymptomatic cases (i.e., individuals who had been infected but showed little or no symptoms) and asymptomatic infection of influenza (i.e., infection caused by an asymptomatic case) do indeed occur regularly (e.g., [14-17]).

Model with only asymptomatic infections, by either asymptomatic or subclinical infectives, during their infectivity period had been recently studied in [14]. In this current study, we will consider a traditional compartmental model which incorporates both pre-symptomatic and asymptomatic infections, in order to explore the role which they could play in the overall spread of disease, if any. Moreover, the age-group structure of the model, by dividing the population into seven groups of the very young, preschool children, younger and older schoolchildren, young adults, adults, and the elderly, allows us to study targeted public health policies (e.g., immunization) aimed at different age groups. Our model also allows for inclusion of immunity and other age-dependent intervention measures such as quarantine and voluntary home withdrawal (see e.g., [15,16]). A full model will be proposed to take into account of the above-mentioned factors that may be important in determining the best vaccine strategy.

## Methods

### Model Formulation

**Figure 1:**
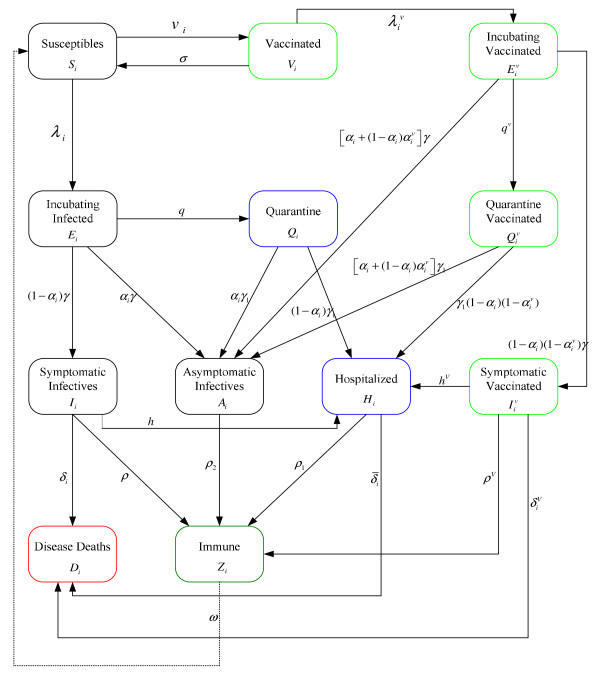
**Model flow diagram**.

Our model is an age-dependent compartmental model. The model flowchart is given in Fig. 1, where the subscript i denotes the i^th ^age group. The model variables are described as follows, with the time unit t in days:

*S_i_(t)*: number of susceptible individuals of the *i*th age group at time *t*;

*V_i_(t)*: number of vaccinated individuals of the *i*th age group at time *t*;

*E_i_(t)*: number of exposed (infected) individuals of the *i*th age group at time *t*;

: number of exposed (infected) vaccinated individuals of the *i*th age group at time *t*;

*Q_i_(t)*: number of quarantined infected individuals of the *i*th age group at time *t*;

*I_i_(t)*: number of infective individuals of the *i*th age group at time *t*;

*I_i_^v^(t)*: number of vaccinated infective individuals of the *i*th age group at time *t*;

*A_i_(t)*: number of asymptomatic (subclinical) infective individuals of the *i*th age group at time *t*;

*H_i_(t)*: number of hospitalized (treated) individuals of the *i*th age group at time *t*;

*Z_i_(t)*: number of recovered and immune individuals of the *i*th age group at time *t*;

*D_i_(t)*: cumulative number of influenza deaths of the *i*th age group at time *t*;

c_ij_: contact rate of an individual of *i*th group with an individual of *j*th group;

*β_ij_*: per contact transmission probability of a susceptible individual of *i*th group by an infective of *j*th group.

*π_i_*: age-specific vaccine efficacy for age group *i*.

*λ_i_(t) *and : disease incidence rates for the susceptible and vaccinated individuals of age group i. See [Additional file 1] for detailed formulae.

The rest of the model parameters are listed in Tables 1-2, with the age-specific parameters given in Table 2. The detailed description of the model is given in [Additional file 1]. Our main model assumptions are as follows:

**Table 1:** Model parameters with parameter values taken from published literature [15,19,21]. The parameter values without a source (mostly 0) are assumed values.

Parameter	Description	Baseline value	Source
*σ*	mean vaccine waning rate	0	
*1/γ*	mean incubation period of exposed individuals	1.48	[21]
*1/γ_1_*	mean time to onset of those who had been quarantined	NA	
*ρ*	mean recovery rate of untreated infectives	1/2.85	[19]
*ρ^V^*	mean recovery rate of vaccinated infectives with *ρ^V ^*≥ *ρ*	1/2.85	[19]
*ρ_2_*	mean recovery rate of asymptomatic infectives	1/2.85	[19]
*ω*	mean immune waning rate	0	
*ϵ*	migration rate of the population	0	
*ϵ_1_*	migration rate of symptomatic infectives	0	
*θ*	immigration rate of the population	0	
*θ_1_*	immigration rate of symptomatic infectives	0	
*q*	quarantine rate of unvaccinated exposed individuals	0	
*q^V^*	quarantine rate of vaccinated exposed individuals	0	
1-ϕ	home withdrawal rate of untreated symptomatic infectives	0	
1-ϕ_1_	home withdrawal rate of all "well" individuals	0	
*τ*	reduction in infectivity of unvaccinated pre-symptomatic infectives	0.4	
*τ_1_*	reduction in infectivity of asymptomatic infectives	0.5	[15]
*τ_2_*	reduction factor in contact due to hospital isolation	0	
*κ*	reduction in infectivity of vaccinated infectives	0.5	[15]

**Table 2:** Age-specific model parameters with the following sources: the age-specific values of vaccine efficacy and effectiveness [8,9], fraction of the symptomatic infectives [15] and [28], and all other values from 2003-2006 Taiwan flu monitor surveillance data.

parameter\age group	0-2	3-5	6-7	8-14	15-21	22-64	≥ 65
time-dependent vaccination rate *ν_i_(t)*	*υ_1_(t)*	0	0	0	0	0	*υ_7_(t)*
vaccine efficacy *π_i _*[8,9]	0.7	0.7	0.7	0.7	0.5	0.5	0.4
fraction of asymptomatic infectives *α_i _*[15,28]	0.5	0.5	0.5	0.5	0.5	0.5	0.7
reduced fraction of asymptomatic infectives due to vaccination [8,9]	0.4	0.4	0.4	0.4	0.4	0.4	0.6
mortality rate of untreated infectives *δ_i_*	6 × 10^-4^	5 × 10^-4^	5 × 10^-5^	5 × 10^-5^	5 × 10^-5^	1 × 10^-4^	0.002
mortality rate of untreated vaccinated infectives *	6 × 10^-4^	5 × 10^-4^	5 × 10^-5^	5 × 10^-5^	5 × 10^-5^	1 × 10^-4^	0.002
mortality rate of hospitalized infectives	0.006	0.005	5 × 10^-4^	5 × 10^-4^	5 × 10^-4^	0.001	0.02
mean recovery rate of the hospitalized infectives *ρ_1_*	1/4.85	1/4.72	1/4.45	1/4.56	1/5.55	1/9.16	1/16.94
**hospitalization rate of unvaccinated symptomatic infectives ***h_i_*	**0.007**	**0.007**	**0.007**	**0.007**	**0.007**	**0.007**	**0.09**
**hospitalization rate of vaccinated symptomatic infectives ***	**0.007**	**0.007**	**0.007**	**0.007**	**0.007**	**0.007**	**0.06**
**per contact transmission probability of infectees in i^th ^group ***β*_*ij *_≡ *β*_*i*_	**0.047**	**0.039**	**0.031**	**0.031**	**0.047**	**0.045**	**0.155**
**per contact transmission probability of infectors in i^th ^group ***β*_*ij *_≡ *β*_*j*_	**0.083**	**0.029**	**0.045**	**0.008**	**0.040**	**0.056**	**0.078**

*Note that *h*_*i *_≥  and  ≤ *δ*_*i*_.

The last four rows (in bold) were obtained by least-squared curve-fitting with data using MATLAB software.

(1) Exposed individuals are infective during the incubation period. It is commonly known (e.g., [18]) that the pre-symptomatic (exposed) individuals cannot transmit the disease in the noninfectious latent period, during which the viral titres gradually increase to detectable and transmissible levels when they became infective for only a short period (0.25 days in [19]) before the onset of symptoms. To avoid adding an extra compartment to account for the (short) period of infectivity after the end of the latent period and before the end of incubation period (see e.g., [20]), we assume that the individuals are infective during the incubation period with the infectivity averaged out over the whole incubation period. This simplification is reasonable since the incubation period for influenza is typically very short, e.g., 1.48 days in [21].

(2) Influenza vaccine is efficacious in preventing influenza infection and effective against influenza-like illness, albeit at different levels of efficacy and effectiveness for different age groups [8,9]. Moreover, the vaccinated individuals are less infectious, once they become infected, when compared to those who had not been vaccinated.

(3) The quarantined individuals will be hospitalized directly following the onset of symptoms (see [22,23] for modeling of quarantine for 2003 SARS outbreak).

(4) A fraction of the infectives has no symptoms or only subclinical symptoms, and is classified as asymptomatic infectives with reduced infectivity [14].

(5) A hospitalized person is removed from isolation either by death or discharged due to recovery from illness.

(6) Homogeneous mixing within subpopulations is assumed.

(7) Negligible births and deaths (excluding disease deaths) during the course of the disease outbreak are assumed.

### Reproduction Numbers

The basic reproduction number *R_0_*, the average number of infections by an infective in an immunologically naive population (see, e.g., Diekmann et al. [24] or van den Driessche and Watmough [25]), is an important epidemiological quantity which gives indication to the potential severity of an epidemic. More precisely, the epidemic cannot be eradicated without interventions if R_0 _exceeds unity. Denoting *S*_0*i *_= *S_i_*(0), we have(1)

where *R_11_*, the average number of infections in group *j *caused by an infective from group *i *in an immunologically naive population, is given by(2)

**Figure 2:**
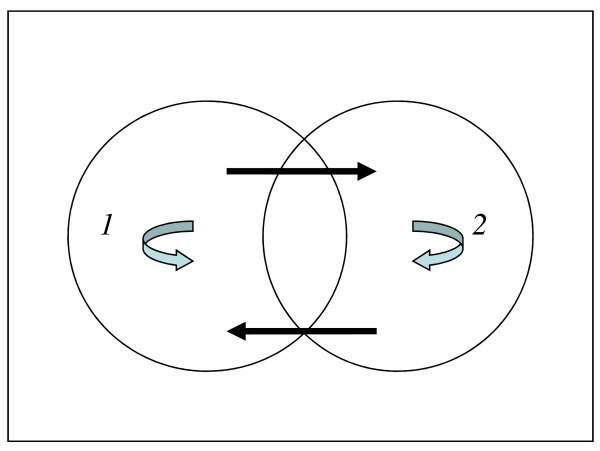
**Graphical illustration of the basic reproduction number for n = 2**. The two blue arrows denotes within-group infections (*R*_11 _and *R*_22_) and the black arrows denote the inter-group infection cycle (*R*_12_*R*_21_). The term *R*_11_*R*_22 _subtracted in (3) accounts the redundancy due to adding both *R*_11 _and *R*_22_.

For illustration, the case n = 2 is described graphically in Fig. 2, where(3)

The two blue arrows denotes within-group infections (*R_11 _*and *R_22_*) and the black arrows denote inter-group infection cycle (*R_12_R_21_*). The term *R_11_R_22 _*subtracted in (3) accounts for the redundancy that resulted when adding *R_11 _*and *R_22_*. Similar results for the basic reproduction number of a multi-group model were also obtained in [26,27].

Moreover, we have the following effective reproduction numbers due to interventions:

(i) The effective reproduction number with interventions excluding vaccination *R_E _*is(4)

*i*, *j *= 1, 2, ..., *n*.

(ii) The effective reproduction number with vaccination only over a time period of immunization [0, T], *R_V_*, is:(5)

where(6)

and

*i*, *j *= 1, 2, ..., *n*, is the average vaccine coverage of age group *i *over the time period [0, T], and(7)

(iii) The effective reproduction number with interventions including vaccination *R_VE _*over the time period [0, T], is(8)

Detailed derivations of the reproduction numbers are also given in [Additional file 1]. Model fit using 2005-2006 Taiwan winter seasonal influenza data and age-specific vaccination data as it was implemented during that flu season, as well as simulation studies of hypothetical scenarios, will be carried out.

### Simulations with Taiwan Seasonal Influenza

With the issue of morbidity-based vs. mortality-based vaccine strategy for pandemic influenza still open to debate, the Taiwan Centers for Disease Control (TCDC) launched a new program of free flu vaccination for 1st and 2nd grades elementary school students (age 6-7) prior to the 2007-2008 winter flu season which was expanded further to include grades 1-4 in the fall of 2008. The aim of this vaccine program is hopefully to lower the seasonal influenza incidence across all age groups of the population. In anticipation of future investigation on public health impact of this program, we carry out model simulations by dividing the Taiwan population into 7 age groups (see Table 2), and by making use of the weekly Taiwan influenza vaccination data for young children of age 2 or less (age group 1, with free flu vaccination since 2004) and the elderly of 65 or older (age group 7, with free flu vaccination since 2001) during the 2004-2005 winter flu season. The average vaccine coverages of age groups 1 and 7 during the 2004-2005 winter season in Taiwan are 63.4% and 58.2%, respectively.

There were no other nonpharmaceutical intervention measures during this winter flu season. That is, all parameters pertaining to quarantine and home withdrawal in the model are set to be 0 in Table 1. Moreover, for the sake of simplicity, we assume no noticeable level of migration and no waning of immunity during the flu season. The rest of the parameter values used are given in Tables 1-2. We also assume a conservative 20% pre-epidemic immunity in our simulation based on a recent sero-epidemiological survey conducted during 2005-2006 winter flu season in Taiwan [28].

For the contact rates between different age groups, we make use of the age-specific contact matrix obtained by Wallinga et al. [29] for Utrecht, the Netherlands, 1986. We adjust for the discrepancy in the population age distributions of Taiwan in 2005 and the Netherlands in 1986, by considering the ratios of demographic age structures of Netherlands in 1987 (Appendix Table 1 in [29]) and of Taiwan in 2005 [Additional file 1: Table A1]. The resulting contact matrix, of the average daily number of contacts for each individual in a certain age group with individuals in another age group, is given in [Additional file 1: Table A2].

### Fitting with Seasonal Influenza Data

The age-dependent hospitalization rates and per contact transmission probability in the last four rows (in bold) in Table 2 were obtained by least-squared curve-fitting with the 2004-2005 Taiwan winter P&I (Pneumonia and Influenza) mortality data from October 9, 2004 to March 5, 2005 for age groups 6 (age 22-64) and 7 (>64) using MATLAB software. *ν*_7_(*τ*) and *ν*_1_(*τ*) are piecewise linear (by week) approximations of the respective weekly vaccination data for elderly (>64) and young children in Taiwan during this time period. To simply our data fitting, we first fitted the data by assuming the per contact transmission probability of the infectees in i^th ^group are averaged, i.e., *β*_*ij *_≡ *β*_*i *_(see next to the last row in bold in Table 2).

## Results

**Figure 3:**
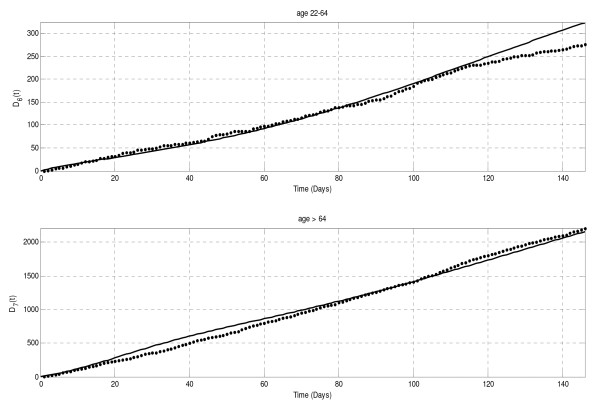
**Model fit for the 2004-2005 (10/9/04-3/5/05) winter cumulative P&I mortality data in Taiwan for age groups 6 (age 22-64) and 7 (>64), with *β*_*ij *_= *β*_*i*_**. The black dots are the real data; the solid curves are simulated *D*_6_(*t*) and *D*_7_(*t*) from the model, where R^2 ^are 0.97222 and 0.99123 for fitting *D*_6_(*t*) and *D*_7_(*t*), respectively.

The result of data fit is given in Fig. 3. The reason for using only groups 6 and 7 for data fit is that the P&I mortality data during that winter season consists mostly of older people. More precisely, 5038 (88.5%) of the 5694 individuals who died of P&I are of age 65 or older (age group 7), 607 (10.7%) are between age 22-64 (age group 6), followed by 17 between age 0-2 (age group 1) and 14 between age 15-21 (age group 5). Each of other age groups has only a handful of cases. Therefore, to avoid large errors due to fitting data with small data size, we only use the data from the two largest groups for our model fit.

**Figure 4:**
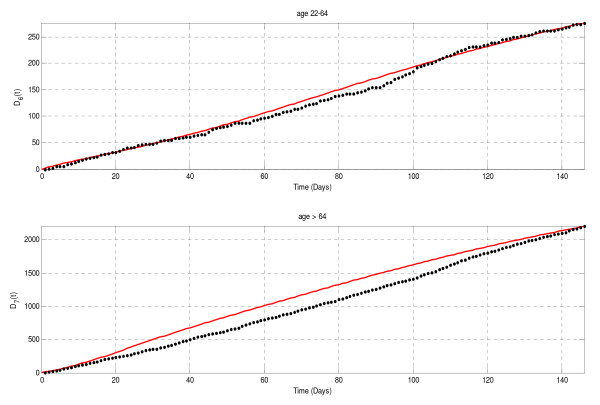
**Model fit for the 2004-2005 (10/9/04-3/5/05) winter cumulative P&I mortality data in Taiwan for age groups 6 (age 22-64) and 7 (>64), with *β*_*ij *_= *β*_*j*_**. The black dots are the real data; the red curves are simulated *D*_6_(*t*) and *D*_7_(*t*) from the model, where R^2 ^are 0.99403 and 0.96763 for fitting *D*_6_(*t*) and *D*_7_(*t*), respectively.

We also fitted the data by assuming the per contact transmission probability of the infectors in i^th ^group are averaged, i.e., *β*_*ij *_≡ *β*_*j *_(see the last row in bold in Table 2 and Fig. 4). We will compare these results to ascertain the difference in age groups on per contact transmission probabilities to and from particular age groups. To give more insight to the data used and the goodness of fit, we also provide the daily observed and predicated mortality corresponding to the case *β*_*ij *_≡ *β*_*j *_in [Additional file 1: Figs. S1-S2].

## **Discussion and Conclusion**

### Age-specific Transmission

By fitting only the hospitalization rates and the per contact transmission probabilities to the 2004-2005 Taiwan winter season P&I mortality for age groups 6 (age 22-64) and 7 (>64), we are able to obtain satisfactory model fit (see Fig. 3), although we note that fitting P&I deaths might conceivably lead to an overestimate of hospitalizations rates. However, simulation studies showed that the model fit is less sensitive to the hospitalization rates than to the transmission probabilities, and intuitively, most sensitive to changes in these rates for the elderly group.

It is interesting to note that comparatively significant errors in both theoretical curves occur in late January roughly after day 110. We note that day 105 was January 22, 2005, when all schools in Taiwan closed for the winter vacation which lasted until after the traditional lunar New Year holiday, in mid-February of that year. The school closure, and subsequent shutdown of all non-essential venues during the week-long New Year holiday, surely had a significant impact on the contact rates which was not reflected in our *constant *contact matrix that implicitly assumes that inter-age group contact patterns remain the same during the whole season. More precisely, the P&I deaths for the elderly exhibits a slight increase for about 3 weeks after the closing of schools (shortly after the New Year) when compared to the model predicted values, while the P&I deaths for the adults of age 22-64 dropped substantially below the theoretical curve. These results indicate that the school closure and the subsequent New Year holiday led to more frequent contacts by the elderly in the households with family members who spent more time at home during the holidays At the same time, there were less contacts for the adults at the workplace (especially for those working in the educational facilities who had longer holidays) during this time period. Since most individuals in the elder group (and the very young) are not directly affected by the school closings and the holidays, one may speculate that the difference in the actual mortality and the theoretical mortality of the elderly, as averaged over the whole time period, is due at least in parts to the impact of interaction between the elderly and younger children with the school-age children and adults, when the activity levels (in terms of frequency as well as whom to make contact with) of the latter groups were significantly changed by the holidays.

Furthermore, the elderly had a higher per contact transmission probability (Table 2) but lower contact frequency [Additional file 1: Table A2]. This study showed that, with the combination of these two factors, morbidity-based vaccination strategy still could be effective for the prevention of elderly mortality. Although a recent study to quantify the effect of school closures during the 2008 winter influenza season in Hong Kong did not find the school closures as having a substantial effect on the community transmission [30], our results indicate the need for further studies on this topic. We also note that, normally, one would like to use averaged excess winter season P&I mortality data over other seasons for the data fitting of seasonal influenza. However, Taiwan often experiences summer influenza epidemics which would offset any attempt to obtain a reasonable "excess" P&I mortality for the winter season.

### The Reproduction Number

Using Equations (2-3) we obtain R_0 _= 1.0001, just above unity. However, we note that it is more appropriately the *reproduction number R *of the winter flu epidemic, given the effect of pre-epidemic immunity that must exist. Chowell et al. [31] used several weekly seasonal flu mortality data, derived from P&I excess deaths and influenza-specific deaths from US, France, and Australia during 1972-1997 (1972-2002 for US), to estimate the (mean) reproduction number R_p _over 3 decades of seasonal flu. They found that the mean of R_p _to be around 1.3, with year-to-year variability of 0.9-2.1. Our estimate is lower, but within their range.

### Vaccination

**Figure 5:**
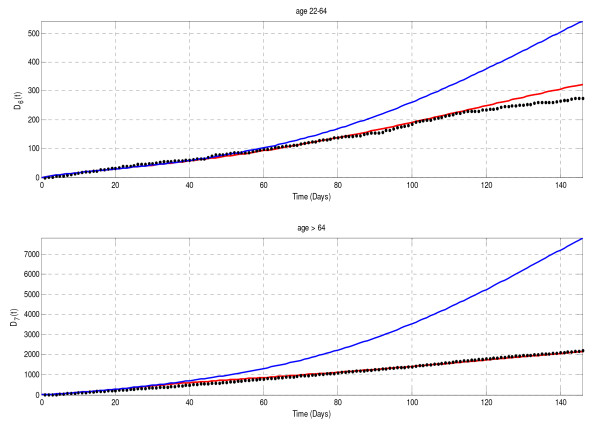
**Simulation with the same model parameters as in Fig. 3, except *υ*_1_(*t*) = *υ*_7_(*t*) = 0**. The black dots are real data, the red curves are model fits in Fig. 3, and the blue curves denote simulation without vaccination.

It is also interesting to note that for our set of parameter values used in Figs. 4, 5, the effective reproduction number with vaccine only (see Equations 5-6) has almost the same value as R_0_, to the fourth decimal digit. One explanation for this apparent lack of impact of vaccination during the 2004-2005 flu season, as indicated by the value of R_V_, is that the vaccination data we used is only for groups 1 (age 0-2) and 7 (age >64). In the formula for R_V_, or more precisely for  in Equation (7), c_ij_^2 ^is the daily contact frequency between groups i and j. The contact frequencies of groups 1 and 7 [Additional file 1: Table A2] are substantially smaller than the contact frequencies of the other groups, with the exception of within-group contacts and contact between groups 1 and 2 (age 3-5) in daycare facilities. Therefore, the relative impact of targeting these two groups for vaccination was weakened by their relative inactivity, thus giving further evidence of the lack of impact of vaccinating the very young and the very old on the overall transmissibility of the disease in the community. However, simulation with the same parameter values as in Fig. 3 except assuming no vaccination in the young children and elderly age groups, i.e., *υ*_1_(*t*) = *υ*_7_(*t*) = 0, and everything else the same produces Fig. 5, where the result indicates that the impact of no vaccination (red curves) would still be significant, resulting in nearly 4-fold increase in mortality of elderly and 2-fold increase in mortality of adults. Therefore, the impact of vaccination on the disease incidence sometimes might not be fully revealed in the change (or the lack thereof) in the effective reproduction number with intervention but could still be substantial, since it is a simple mathematical property that distinctly different (next generation) matrices may have similar eigenvalues.

Furthermore, in our simulations we had assumed vaccine efficacy (proportion of infection prevented) of 40% for elderly and 70% for young children, to be in line with current literature [8,9]. However, efficacy depends very much on matching of the circulating strains with vaccine strains each year, where mismatch often causes low efficacy and might have affected our resulting data fit. We also assumed vaccine effectiveness (proportion of reduction in symptomatic cases) to be 60% for elderly and 40% for young children (also see [8,9]).

The per contact transmission probability for the susceptible elderly (>64) *infectees *(see next to the last row in Table 2) is estimated to be 0.155, more than three-fold of any other age group (with the young children of 0-2 being the next highest), which may be due to the common need for very close contact while the elderly (or younger children of age <3) are being cared for, typically by individuals from adults of ages 22-64 in age group 6, even though the *frequency *of contact might be less than that of with other age groups. This further highlights the high vulnerability of the elderly (or the younger children) to exposure from other age groups, and demonstrates the need for morbidity-based strategy to prevent the elderly (or younger children) influenza mortality. On the other hand, the estimated per contact transmission probabilities for the younger (<3) and the elderly (>64) *infectors *are also both higher than those of the other groups (see the last row in Table 2), but not significantly so except when compared with those between ages 8-14. The higher transmission probabilities of young children and elderly infectives could reflect, again, the fact that contacts with these individuals are usually of a more intimate nature, although these probabilities are not as drastically different as their vulnerability to be infected. The less likelihood of schoolchildren of 8-14 to infect others might also be attributable to signs of their less intimate contacts with others as they grow into adolescence.

To further explore this situation, we note that from Equation (6), the partial derivatives of  with respect to *ν*_*i*_π_*i *_are:

We know  from Equations (2) and (7). We also know that *κ *∈ [0, 1] and *ν*_*i*_π_*i *_for all *i*, *j*. Subsequently,  is a nonincreasing function of *ν*_*i*_π_*i *_for all *i, j*. However, since *R*_*V *_= (-1)^*n*+1 ^det *R*^*V *^+ 1, where  is the matrix with  its *ij*th element, the effective reproduction number with vaccination only, *R_V_*, does not necessarily decrease as the effective vaccination rate *ν*_*i*_π_*i *_increases. In other words, vaccination is not always beneficial in reducing incidence and the design of an effective vaccination program in multi-group model is highly nontrivial, unlike in simple epidemic models where there is a simple formula for the critical vaccination coverage level necessary for eradication (p_c_) [20]. In fact, it has been shown mathematically by Hadeler and Castillo-Chavez [32], using a model with a core group, that partially effective vaccination program may actually increase the total number of cases. Explicit quantification of an optimal vaccination policy ([33]) in a multi-group population scenario remains to be a challenge for mathematical modelers and is beyond the scope of this work. Moreover, the model fit was carried out with vaccination (of the elderly and small children of age 2 or less) as the only intervention since vaccination was the only intervention that was implemented during the flu seasonal for which the fitted P&I data was collected. Further simulations with quarantine and home withdrawal also can be easily carried out.

### Pre-epidemic Immunity

A related issue is that of pre-epidemic immunity. Further sensitivity analysis using pre-immunity in the range of 10%-30% has shown that the results of curve-fittings are not sensitive to small changes in the pre-epidemic immunity. One would expect that pre-epidemic immunity does impact the outbreak, but nonetheless it is not reflected, at least not in the reproduction numbers.

**Figure 6:**
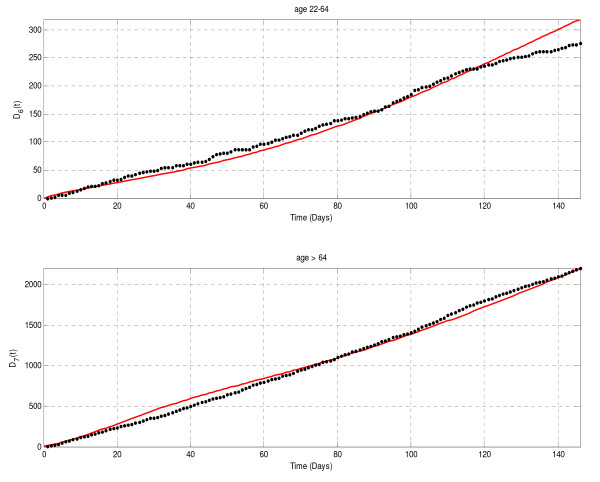
**Model fit with same model parameters as Fig. 3 except no asymptomatic infection by asymptomatic infectives (*τ *= 0)**. The black dots are the real data; the red curves are the model fit, where R^2 ^are 0.97346 and 0.99259, respectively.

**Figure 7:**
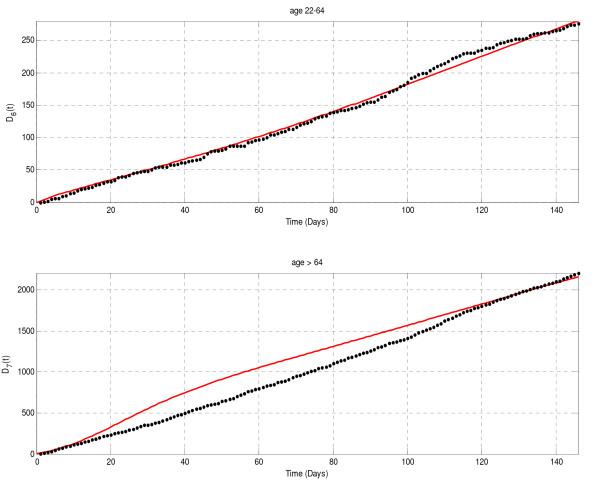
**Model fit with same model parameters as Fig. 3 except no asymptomatic infection by asymptomatic infectives (*αi *= 0)**. The black dots are the real data; the red curves are the model fit, where R^2 ^are 0.99472 and 0.94446, respectively.

Pre-symptomatic infection and asymptomatic infection by asymptomatic infectives were incorporated into the proposed model. To gauge the roles these two features of influenza might play in a seasonal influenza epidemic, we performed the following simulations. Again using Fig. 3 as a benchmark, we first assume no pre-symptomatic infection (*τ *= 0) with all other parameter values unchanged (Fig. 6). Next we assume no asymptomatic infection by asymptomatic infectives (*α*_*i *_= *α*_*i*_^*V *^= 0) (Fig. 7). The results indicate that pre-symptomatic infection seemed to have little effect on the model fits and the fitted parameters; while asymptomatic infection by asymptomatic infectives has a more pronounced impact on the model fit for the elderly, perhaps indicating the comparatively larger role asymptomatic infection plays in disease transmission.

Finally, as the WHO Strategic Advisory Group of Experts (SAGE) recently made its recommendations for priorities in vaccination for the H1N1 pandemic in terms of the social groups (e.g., healthcare workers those with chronic medical conditions), health groups (pregnant women), and age groups [34], the model also can be used to divide population into social/health groups. For example, to study vaccine policy for the elderly, we could divide the elderly into those living in households and those living in old age homes, since they mix differently in these two distinct settings. The model is also useful for simulations of the cost-effectiveness of vaccine and other intervention measures, such as prophylaxis treatment, perhaps in future work.

## Competing interests

The authors declare that they have no competing interests.

## Pre-publication history

The pre-publication history for this paper can be accessed here:

http://www.biomedcentral.com/1471-2334/10/106/prepub

## Supplementary Material

Additional file 1 **Electronic Supplementary Material**. SVEQIAHR Model Details and Supplementary Data. Click here for file
