# Supplementary material for: Age groups and spread of influenza: implications for vaccination strategy
Source: BMC Infect Dis. 2010 Apr 30;10:106. doi: 10.1186/1471-2334-10-106 (PMC2876165; doi:10.1186/1471-2334-10-106)
Supplement: Additional file 1 — Electronic Supplementary Material. SVEQIAHR Model Details and Supplementary Data. [file 1471-2334-10-106-S1.DOC]

**Additional file I**

**Electronic Supplementary Material:**

**SVEQIAHR Model Details and Supplementary Data**

Ying-Hen Hsieh

Department of Public Health and Center of Infectious Disease Epidemiology Research, China Medical University, Taichung, Taiwan

Correspondence to: Dr. Ying-Hen Hsieh, Department of Public Health, China Medical University, Taichung, Taiwan 40402. Tel: 886-4-22053366x6109, Fax: 886-4-22078539, email: [hsieh@mail.cmu.edu.tw](mailto:hsieh@mail.cmu.edu.tw).

**1. SVEQIAHR Model**

The model flowchart is given in Fig. 1, where the subscript i denotes the i^th^ age group. The model is a system of 12×n ordinary differential equations, with n age-groups and i=1,2,…,n. The time unit is in days. The model equations are as follows:

$$\frac{dS_{i}}{dt}=-\left[\nu_{i}(t)+\varepsilon+\lambda_{i}(t)\right]S_{i}(t)+\theta S_{i}(t)+\sigma V_{i}\left(t\right)+\omega Z_{i}(t)$$

$$\frac{dV_{i}}{dt}=\nu_{i}(t)S_{i}(t)-\left[\sigma+\varepsilon+\lambda_{i}^{V}(t)\right]V_{i}\left(t\right)+\theta V_{i}(t)$$

$$\frac{dE_{i}}{dt}=\lambda_{i}(t)S_{i}\left(t\right)-\left[\gamma+\varepsilon+q\right]E_{i}\left(t\right)+\theta E_{i}(t)$$

$$\frac{dE_{i}^{V}}{dt}=\lambda_{i}^{V}(t)V_{i}\left(t\right)-\left[\gamma+\varepsilon+q^{V}\right]E_{_{i}}^{V}\left(t\right)+\theta E_{_{i}}^{V}(t)$$

$$\frac{dQ_{i}}{dt}=qE_{i}\left(t\right)-\gamma_{1}Q_{i}\left(t\right)$$

$$\frac{dQ_{i}^{V}}{dt}=q^{V}E_{i}^{V}\left(t\right)-\gamma_{1}Q_{i}^{V}\left(t\right)$$

$\frac{dI_{i}}{dt}=(1-\alpha_{i})\gamma E_{i}\left(t\right)-\left[h_{i}+\rho+\varepsilon_{1}+\delta_{i}\right]I_{i}\left(t\right)+\theta_{1}I_{i}(t)$ (1)

$$\frac{dI_{_{i}}^{V}}{dt}=(1-\alpha_{i})(1-\alpha_{i}^{V})\gamma E_{_{i}}^{V}\left(t\right)-\left[h_{i}^{V}+\rho^{V}+\varepsilon_{1}+\delta_{i}^{V}\right]I_{_{i}}^{V}\left(t\right)+\theta_{1}I_{_{i}}^{V}(t)$$

$$\begin{matrix} \frac{dA_{i}}{dt}=\alpha_{i}[\gamma E_{i}\left(t\right)+\gamma_{1}Q_{i}\left(t\right)]+\gamma[\alpha_{i}+\alpha_{i}^{V}(1-\alpha_{i})]E_{i}^{V}(t)+\gamma_{1}[\alpha_{i}+\alpha_{i}^{V}(1-\alpha_{i})]Q_{i}^{V}\left(t\right) \\ \text{ }-[\rho_{2}+\varepsilon]A_{i}(t)+\theta A_{i}(t) \end{matrix}$$

$$\frac{dH_{i}}{dt}=h_{i}I_{i}\left(t\right)+(1-\alpha_{i})\gamma_{1}Q_{i}(t)+(1-\alpha_{i})(1-\alpha_{i}^{V})\gamma_{1}Q_{i}^{V}(t)-[\rho_{1}+\overline{\delta}_{i}]H_{i}(t)+h_{i}^{V}I_{_{i}}^{V}\left(t\right)$$

$$\frac{dZ_{i}}{dt}=\rho I_{i}\left(t\right)+\rho_{1}H_{i}\left(t\right)+\rho_{2}A_{i}\left(t\right)-\left[\omega+\varepsilon\right]Z_{i}\left(t\right)+\theta Z_{i}(t)+\rho^{V}I_{_{i}}^{V}\left(t\right)$$

$$\frac{dD_{i}}{dt}=\delta_{i}I_{i}\left(t\right)+\overline{\delta}_{i}H_{i}(t)+\delta_{i}^{V}I_{_{i}}^{V}\left(t\right).$$

The frequency-dependent incidence rates for the susceptible and vaccinated individuals of age group i, $\lambda_{i}(t)\text{ and }\lambda_{i}^{V}(t)$, are given by the respective formulae $\lambda_{i}\text{(t) =}\sum_{j=1}^{n} c_{ij}\beta_{ij}\frac{c_{ij}[\phi I_{j}(t)\text{+}\tau\phi_{1}E_{j}(t)\text{+}\tau_{1}\phi_{1}A_{j}(t)+\tau_{2}H_{j}(t)+\phi\kappa I_{_{j}}^{V}(t)+\tau\phi_{1}E_{_{j}}^{V}(t)]}{N_{i}\text{(t)}}$, (2a)

$\lambda_{i}^{V}\text{(t) =}\sum_{j=1}^{n} c_{ij}\beta_{ij}(1-\pi_{i})\frac{c_{ij}[\phi\kappa I_{_{j}}^{V}(t)+\tau\phi_{1}E_{_{j}}^{V}(t)+\phi I_{j}(t)\text{+}\tau\phi_{1}E_{j}(t)\text{+}\tau_{1}\phi_{1}A_{j}(t)+\tau_{2}H_{j}(t)]}{N_{i}\text{(t)}},$ (2b)

where $N_{i}(t)=\sum_{j=1}^{n} c_{ij}[\phi_{1}(S_{j}+V_{j}+A_{j}+E_{j}+E_{_{j}}^{V})+\phi(I_{j}+I_{j}^{V})]$ is the total number of contacts by an individual of age group i at time t.

The model variables are described as follows:

$S_{i}\left(t\right)$: number of susceptible individuals of the *i*th age group at time *t*;

$V_{i}\left(t\right)$: number of vaccinated individuals of the *i*th age group at time *t*;

$E_{i}\left(t\right)$: number of exposed (infected) individuals of the *i*th age group at time *t*;

$E_{i}^{V}\left(t\right)$: number of exposed (infected) vaccinated individuals of the *i*th age group at time *t*;

$Q_{i}\left(t\right)$: number of quarantined infected individuals of the *i*th age group at time *t*;

$I_{i}\left(t\right)$: number of infective individuals of the *i*th age group at time *t*;

$I_{i}^{V}\left(t\right)$: number of vaccinated infective individuals of the *i*th age group at time *t*;

$A_{i}\left(t\right)$: number of asymptomatic (subclinical) infective individuals of the *i*th age group at time *t*;

$H_{i}\left(t\right)$: number of hospitalized (treated) individuals of the *i*th age group at time *t*;

$Z_{i}\left(t\right)$: number of recovered and immune individuals of the *i*th age group at time *t*;

$D_{i}\left(t\right)$: number of death individuals of the *i*th age group at time *t*;

c_ij_: contact rate of an individual of *i*th group with an individual of *j*th group;

$\beta_{ij}$: per contact transmission probability of a susceptible individual of *i*th group by an infective of *j*th group;

*π_i_* : age-specific vaccine efficacy for age group i.

The other model parameters are listed in Table 1.

**2. Reproduction Numbers**

First we consider the case at the initial stage with no intervention (vaccination, quarantine, home withdrawal, etc.), and thus obtain the following equations:

$\begin{matrix} \frac{dE_{i}}{dt}=\lambda_{i}S_{i}-\left(\gamma+\varepsilon\right)E_{i}+\theta E_{i} \\ \frac{dI_{i}}{dt}=\left(1-\alpha_{i}\right)\gamma E_{i}-\left(h_{i}+\rho+\varepsilon_{1}+\delta_{i}\right)I_{i}+\theta_{1}I_{i} \\ \frac{dA_{i}}{dt}=\alpha_{i}\left(\gamma E_{i}\right)-\left(\rho_{2}+\varepsilon\right)A_{i}+\theta A_{i} \\ \frac{dH_{i}}{dt}=h_{i}I_{i}-\left(\rho_{1}+\delta_{i}\right)H_{i}, \end{matrix}$ (3)

where i=1,2,…,n, and $\lambda_{i}\text{(t) =}\sum_{j=1}^{n} c_{ij}\beta_{ij}\frac{c_{ij}[\text{I}_{\text{j}}\text{(t)+}\tau E_{j}(t)\text{+}\tau_{1}A_{j}(t)+\tau_{2}H_{j}(t)]}{N_{i}\text{(t)}}$.

For brevity, we also redefine $\bar{\gamma}$ to be the former $\gamma+\varepsilon-\theta$, $\bar{\rho}_{i}$ to be the former $h_{i}+\rho+\varepsilon_{1}+\delta_{i}-\theta_{1}$, $\rho_{i}^{*}$ to be the former $\rho_{1}+\overline{\delta}_{i}$, $\widetilde{\rho}_{2}$ to be the former $\rho_{2}+\varepsilon-\theta$, and the equations are simplified to

$\begin{matrix} \frac{dE_{i}}{dt}=\lambda_{i}S_{i}-\bar{\gamma}E_{i} \\ \frac{dI_{i}}{dt}=\left(1-\alpha_{i}\right)\gamma E_{i}-\bar{\rho}_{i}I_{i} \\ \frac{dA_{i}}{dt}=\alpha_{i}\gamma E_{i}-\widetilde{\rho}_{2}A_{i} \\ \frac{dH_{i}}{dt}=h_{i}I_{i}-\rho_{i}^{*}H_{i}. \end{matrix}$ (4)

Denoting $S_{0i}=S_{i}(0)$, the basic reproduction number *R_0_* (see, e.g., Diekmann et al. [8] or van den Driessche and Watmough [9]) for disease-free equilibrium (DFE) is

$R_{0}=\left(-1\right)^{n+1}\det\left(\begin{matrix} R_{11}-1 & R_{12} & \cdots& R_{1n} \\ R_{21} & R_{22}-1 & \cdots& R_{2n} \\ \vdots& \vdots& \ddots& \vdots\\ R_{n1} & R_{n2} & \cdots& R_{nn}-1 \end{matrix}\right)+1$ (5)

where

$R_{ij}=\frac{c_{ij}^{2}\beta_{ij}S_{0i}}{\sum_{k=1}^{n} c_{ik}S_{0k}\bar{\gamma}}\left[\tau+\frac{\gamma\left(1-\alpha_{j}\right)}{\bar{\rho}_{j}}+\frac{\tau_{1}\alpha_{j}\gamma}{\widetilde{\rho}_{2}}+\frac{\tau_{2}\gamma h_{j}\left(1-\alpha_{j}\right)}{\bar{\rho}_{j}\rho_{j}^{*}}\right]$, $i,j=1,2,\cdots,n$. (6)

**2.1. Effective Reproduction Number with Interventions**

Again, for brevity, the equations are simplified to

$\begin{matrix} \text{ }\frac{dE_{i}}{dt}=\lambda_{i}S_{i}-\overline{\gamma}E_{i}-qE_{i}\left(t\right) \\ \frac{dQ_{i}}{dt}=qE_{i}\left(t\right)-\gamma_{1}Q_{i}\left(t\right) \\ \frac{dA_{i}}{dt}=\alpha_{i}[\gamma E_{i}+\gamma_{1}Q_{i}]-\widetilde{\rho}_{2}A_{i} \\ \frac{dI_{i}}{dt}=(1-\alpha_{i})\gamma E_{i}-\bar{\rho}_{i}I_{i} \\ \frac{dH_{i}}{dt}=h_{i}I_{i}+(1-\alpha_{i})\gamma_{1}Q_{i}-\rho_{i}^{*}H_{i}. \end{matrix}$ (7)

We have the following effective reproduction numbers:

(i) The effective reproduction number with interventions other than vaccination *R_E_* is

$R_{E}=\left(-1\right)^{n+1}\det\left(\begin{matrix} R_{11}^{E}-1 & R_{12}^{E} & \cdots& R_{1n}^{E} \\ R_{21}^{E} & R_{22}^{E}-1 & \cdots& R_{2n}^{E} \\ \vdots& \vdots& \ddots& \vdots\\ R_{n1}^{E} & R_{n2}^{E} & \cdots& R_{nn}^{E}-1 \end{matrix}\right)+1$, where

$R_{ij}^{E}=\frac{c_{ij}^{2}\beta_{ij}S_{0i}}{\sum_{k=1}^{n} c_{ik}S_{0k}(\bar{\gamma}+q)}\left[\tau+\frac{\gamma\phi\left(1-\alpha_{j}\right)}{\phi_{1}\bar{\rho}_{j}}+\frac{\tau_{1}\alpha_{j}\gamma}{\widetilde{\rho}_{2}}+\frac{\tau_{1}\alpha_{j}q}{\widetilde{\rho}_{2}}+\frac{\tau_{2}\gamma h_{j}\left(1-\alpha_{j}\right)}{\phi_{1}\bar{\rho}_{j}\rho_{j}^{*}}+\frac{\tau_{2}q\left(1-\alpha_{j}\right)}{\phi_{1}\rho_{j}^{*}}\right]$, (8) $i,j=1,2,\cdots,n$.

(ii) For model with vaccination but no other nonpharmaceutical interventions, $\overline{\rho}_{i}^{V}=h_{i}^{V}+\rho^{V}+\varepsilon_{1}+\delta_{i}^{V}-\theta_{1}$. We have

$$\frac{dE_{i}}{dt}=\lambda_{i}(t)S_{i}\left(t\right)-\overline{\gamma}E_{i}\left(t\right)$$

$$\frac{dE_{i}^{V}}{dt}=\lambda_{i}^{V}(t)V_{i}\left(t\right)-\overline{\gamma}E_{_{i}}^{V}\left(t\right)$$

$\frac{dA_{i}}{dt}=\alpha_{i}\gamma E_{i}\left(t\right)+\gamma[\alpha_{i}+\alpha_{i}^{V}(1-\alpha_{i})]E_{i}^{V}(t)-\widetilde{\rho}_{2}A_{i}(t)$ (9)

$$\frac{dI_{i}}{dt}=(1-\alpha_{i})\gamma E_{i}\left(t\right)-\overline{\rho}_{i}I_{i}\left(t\right)$$

$$\frac{dI_{_{i}}^{V}}{dt}=(1-\alpha_{i})(1-\alpha_{i}^{V})\gamma E_{_{i}}^{V}\left(t\right)-\overline{\rho}_{i}^{V}I_{_{i}}^{V}\left(t\right)$$

$$\frac{dH_{i}}{dt}=h_{i}I_{i}\left(t\right)-\rho_{i}^{*}H_{i}(t)+h_{i}^{V}I_{_{i}}^{V}\left(t\right)$$

The effective reproduction number with vaccination only over the time period [0, T], *R_V_* is:

$R_{V}=\left(-1\right)^{n+1}\det\left(\begin{matrix} R_{11}^{V}-1 & R_{12}^{V} & \cdots& R_{1n}^{V} \\ R_{21}^{V} & R_{22}^{V}-1 & \cdots& R_{2n}^{V} \\ \vdots& \vdots& \ddots& \vdots\\ R_{n1}^{V} & R_{n2}^{V} & \cdots& R_{nn}^{V}-1 \end{matrix}\right)+1$, where

$R_{ij}^{V}=[(1-\nu_{i}\pi_{i})R_{ij}+\nu_{i}\pi_{i}R_{ij}^{0V}][1-(1-\kappa)\nu_{j}\pi_{j}]$ (10)

with

$\nu_{i}=\frac{\int_{t=0}^{T} v_{i}(\tau)S_{i}(\tau)dt}{\int_{t=0}^{T} S_{i}(t)dt}$, $i,j=1,2,\cdots,n$

and

$R_{ij}^{0V}=\frac{c_{ij}^{2}\beta_{ij}S_{0i}}{\sum_{k=1}^{n} c_{ik}S_{0k}\bar{\gamma}}\left\{\tau+\frac{\gamma\left(1-\alpha_{j}\right)\left(1-\alpha_{j}^{V}\right)}{\bar{\rho}_{_{j}}^{V}}+\frac{\tau_{1}\gamma[\alpha_{j}+\left(1-\alpha_{j}\right)\alpha_{j}^{V}]}{\widetilde{\rho}_{2}}+\frac{\tau_{2}\gamma h_{_{j}}^{V}\left(1-\alpha_{j}\right)\left(1-\alpha_{j}^{V}\right)}{\bar{\rho}_{j}^{V}\rho_{j}^{*}}\right\}$ (11)

(iii) The effective reproduction number with vaccination *R_VE_* over the time period [0, T], is

$R_{EV}=\left(-1\right)^{n+1}\det\left(\begin{matrix} R_{11}^{EV}-1 & R_{12}^{EV} & \cdots& R_{1n}^{EV} \\ R_{21}^{EV} & R_{22}^{EV}-1 & \cdots& R_{2n}^{EV} \\ \vdots& \vdots& \ddots& \vdots\\ R_{n1}^{EV} & R_{n2}^{EV} & \cdots& R_{nn}^{EV}-1 \end{matrix}\right)+1$, where

$R_{ij}^{EV}=[(1-\nu_{i}\pi_{i})R_{ij}^{E}+\nu_{i}\pi_{i}\overline{R}_{ij}^{V}][1-(1-\kappa)\nu_{j}\pi_{j}]$ with

$\overline{R}_{ij}^{V}=\frac{c_{ij}^{2}\beta_{ij}S_{0i}}{\sum_{k=1}^{n} c_{ik}S_{0k}(\bar{\gamma}+q^{V})}\{\tau+\frac{\gamma\phi\left(1-\alpha_{j}\right)\left(1-\alpha_{j}^{V}\right)}{\phi_{1}\bar{\rho}_{_{j}}^{V}}+\frac{\tau_{1}\gamma[\alpha_{j}+\left(1-\alpha_{j}\right)\alpha_{j}^{V}]}{\widetilde{\rho}_{2}}$ $+\frac{\tau_{1}[\alpha_{j}+\left(1-\alpha_{j}\right)\alpha_{j}^{V}]q^{V}}{\widetilde{\rho}_{2}}+\frac{\tau_{2}\gamma h_{_{j}}^{V}\left(1-\alpha_{j}\right)\left(1-\alpha_{j}^{V}\right)}{\phi_{1}\bar{\rho}_{_{j}}^{V}\rho_{j}^{*}}+\frac{\tau_{2}q^{V}\left(1-\alpha_{j}\right)\left(1-\alpha_{j}^{V}\right)}{\phi_{1}\rho_{j}^{*}}\}.$ (13)

Table A1. 2005 Taiwan population age structure.

| Age groups | Age | population | % |
| --- | --- | --- | --- |
| 1 | 0-2 | 641095 | 2.82 |
| 2 | 3-5 | 809663 | 3.56 |
| 3 | 6-7 | 550564 | 2.42 |
| 4 | 8-14 | 2257727 | 9.92 |
| 5 | 15-21 | 2286293 | 10.04 |
| 6 | 22-64 | 14008237 | 61.52 |
| 7 | ≥ 65 | 2216804 | 9.74 |
| Total | - | 22770383 | 100.00 |

Table A2. Contact matrix [c_ij_] used in simulation, obtained from [10] by adjusting for the difference in age distribution between Netherlands 1986 and Taiwan 2005. c_ij_ is the daily number of contacts for each individual in age group i with individuals in age group j.

| Age group | 0-2 | 3-5 | 6-7 | 8-14 | 15-21 | 22-64 | ≥ 65 |
| --- | --- | --- | --- | --- | --- | --- | --- |
| 0-2 | 26.64 | 26.64 | 4.97 | 4.26 | 6.39 | 8.88 | 2.84 |
| 3-5 | 26.44 | 26.44 | 4.78 | 4.50 | 6.47 | 8.72 | 2.53 |
| 6-7 | 4.96 | 4.96 | 37.64 | 28.54 | 5.79 | 6.62 | 2.07 |
| 8-14 | 4.34 | 4.34 | 28.34 | 24.10 | 12.30 | 7.36 | 2.22 |
| 15-21 | 6.37 | 6.37 | 5.98 | 12.15 | 23.40 | 9.46 | 3.49 |
| 22-64 | 8.76 | 8.76 | 6.47 | 7.40 | 9.14 | 7.17 | 5.66 |
| ≥ 65 | 2.67 | 2.67 | 2.05 | 2.26 | 3.49 | 5.65 | 10.37 |

**References**

1. Baccam P, Beauchemin C, Macken CA, Hayden FG, Perelson AS: **Kinetics of influenza A virus infection in humans.** *J Virol* 2006, **80**:7590–7599.
2. [Alexander ME](http://www.ncbi.nlm.nih.gov/sites/entrez?Db=pubmed&Cmd=Search&Term="Alexander ME"%5BAuthor%5D&itool=EntrezSystem2.PEntrez.Pubmed.Pubmed_ResultsPanel.Pubmed_DiscoveryPanel.Pubmed_RVAbstractPlus), [Bowman CS](http://www.ncbi.nlm.nih.gov/sites/entrez?Db=pubmed&Cmd=Search&Term="Bowman CS"%5BAuthor%5D&itool=EntrezSystem2.PEntrez.Pubmed.Pubmed_ResultsPanel.Pubmed_DiscoveryPanel.Pubmed_RVAbstractPlus), [Feng Z](http://www.ncbi.nlm.nih.gov/sites/entrez?Db=pubmed&Cmd=Search&Term="Feng Z"%5BAuthor%5D&itool=EntrezSystem2.PEntrez.Pubmed.Pubmed_ResultsPanel.Pubmed_DiscoveryPanel.Pubmed_RVAbstractPlus), [Gardam M](http://www.ncbi.nlm.nih.gov/sites/entrez?Db=pubmed&Cmd=Search&Term="Gardam M"%5BAuthor%5D&itool=EntrezSystem2.PEntrez.Pubmed.Pubmed_ResultsPanel.Pubmed_DiscoveryPanel.Pubmed_RVAbstractPlus), [Moghadas SM](http://www.ncbi.nlm.nih.gov/sites/entrez?Db=pubmed&Cmd=Search&Term="Moghadas SM"%5BAuthor%5D&itool=EntrezSystem2.PEntrez.Pubmed.Pubmed_ResultsPanel.Pubmed_DiscoveryPanel.Pubmed_RVAbstractPlus), [Röst G](http://www.ncbi.nlm.nih.gov/sites/entrez?Db=pubmed&Cmd=Search&Term="Röst G"%5BAuthor%5D&itool=EntrezSystem2.PEntrez.Pubmed.Pubmed_ResultsPanel.Pubmed_DiscoveryPanel.Pubmed_RVAbstractPlus), [Wu J](http://www.ncbi.nlm.nih.gov/sites/entrez?Db=pubmed&Cmd=Search&Term="Wu J"%5BAuthor%5D&itool=EntrezSystem2.PEntrez.Pubmed.Pubmed_ResultsPanel.Pubmed_DiscoveryPanel.Pubmed_RVAbstractPlus), [Yan P](http://www.ncbi.nlm.nih.gov/sites/entrez?Db=pubmed&Cmd=Search&Term="Yan P"%5BAuthor%5D&itool=EntrezSystem2.PEntrez.Pubmed.Pubmed_ResultsPanel.Pubmed_DiscoveryPanel.Pubmed_RVAbstractPlus): **Emergence of drug resistance: implications for antiviral control of pandemic influenza.** *Proc Biol Sci* 2007, **274**(1619): 1675–1684.
3. Jefferson T, Smith S, Demicheli V, Harnden A, Rivetti A, Pietrantonj C Di: **Assessment of the efficacy and effectiveness of influenza vaccines in healthy children: systematic review.** *Lancet* 2005, **365**:773–780.
4. Jefferson T, Rivetti D, Rivetti A, Rudin M, Pietrantonj C Di, Demicheli V : **Efficacy and effectiveness of influenza vaccines in elderly people: a systematic review.** *Lancet* 2005, **366**:1165–1174.
5. Hsieh YH, King CC, Ho MS, Chen CWS, Lee JY, Liu FC, Wu YC, Wu JSJ: **Quarantine for SARS, Taiwan.** *Emerg Infect Dis* 2005, **11**(2):278–282.
6. Hsieh YH, King CC, Chen CWS, Ho MS, Hsu SB, Wu YC: **Impact of Quarantine on the 2003 SARS Outbreak: a retrospective modeling study**. *J Theoretical Biology* 2007, **244**:729–736.
7. Hsu SB, Hsieh YH: **On the Role of Asymptomatic Infection in Transmission Dynamics of Infectious Diseases**. *Bull Math Biology* 2008, **70**:134–155.
8. Diekmann O, Heesterbeek JAP, Metz JAJ: **On the definition and the computation of the basic reproduction ratio R_0_ in models for infectious diseases in heterogeneous populations**. *J Math Biol* 1990, **28**:365–382.
9. van den Driessche P, Watmough J: **Reproduction numbers and sub-threshold endemic equilibria for compartmental models of disease transmission.** *Math Biosci* 2002, **180**:29–48.
10. Wallinga J, Teunis P, Kretzschmar M: **Using data on social contacts to estimate age-specific transmission parameters for respiratory-spread infectious agents**. *Am J Epidemiol* 2006, **164**: 936-944.

Fig. S1. Daily observed and predicated mortality for age group 6 corresponding to the case $\beta_{ij}\equiv\beta_{j}$ (last row in bold in Table 2 and Fig. 4).

Fig. S2. Daily observed and predicated mortality for age group 7 corresponding to the case $\beta_{ij}\equiv\beta_{j}$ (last row in bold in Table 2 and Fig. 4).
